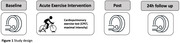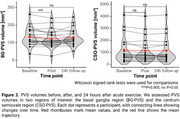# Acute physical exercise can exert measurable changes on perivascular spaces volumetry

**DOI:** 10.1002/alz70855_101885

**Published:** 2025-12-23

**Authors:** Christiane Piechowiak, Patrick Müller, Jose Bernal Moyano, Naomi Alice Kunz, Suzann Al‐Zawity, Patrick Hubert, Brigitta Patricia Nathania, Matthias Kunz, Yves Lading, Roberto Duarte, Maria del C. Valdes Hernandez, Joanna M Wardlaw, Hendrik Mattern, Daniel Behme, Katja Neumann, Rüdiger Braun‐Dullaeus, Stefanie Schreiber

**Affiliations:** ^1^ University Hospital Magdeburg, Magdeburg, Sachsen‐Anhalt, Germany; ^2^ University Hospital Magdeburg, Magdeburg, Germany; ^3^ German Center for Neurodegenerative Diseases (DZNE), Magdeburg, Germany; ^4^ German Center for Mental Health (DZPG), Magdeburg, Germany; ^5^ Center for Intervention and Research on adaptive and maladaptive brain Circuits underlying mental health (C‐I‐R‐C), Magdeburg, Germany; ^6^ Centre for Clinical Brain Sciences, The University of Edinburgh, Edinburgh, Scotland, United Kingdom; ^7^ University of Edinburgh and UK DRI, Edinburgh, United Kingdom; ^8^ Institute of Cognitive Neurology and Dementia Research (IKND), Otto‐von‐Guericke University, Magdeburg, Germany; ^9^ Department of Biomedical Magnetic Resonance, Otto‐von‐Guericke University, Magdeburg, Germany; ^10^ Department of Neuroradiology, University Hospital Magdeburg, Magdeburg, Sachsen‐Anhalt, Germany; ^11^ Department of Neurology, Otto‐von‐Guericke University, Magdeburg, Germany; ^12^ Center for Behavioural Brain Sciences (CBBS), Magdeburg, Sachsen‐Anhalt, Germany

## Abstract

**Background:**

Physical activity has been shown to reduce the risk of dementia and the pathological accumulation of amyloid in both animals and humans. One potential explanation for this outcome is that physical activity enhances glymphatic function. In this study we investigated whether a single session of physical exercise, could alter the glymphatic system, operationalized here as the visibility of perivascular spaces (PVS) on magnetic resonance imaging (MRI).

**Method:**

In this prospective cohort study, we included 20 young participants (mean age 25.8±3.5 years, female 50%), who underwent repeated MRI scans at three different time points: baseline, immediately after cardiopulmonary exercise testing until exhaustion, and 24 hours later (Figure 1). We estimated PVS volumes in the centrum semiovale (CSO) and basal ganglia (BG) using a well‐validated software. For each subject, we first aligned all T2‐weighted images using FreeSurfer's mri_robust_template tool. Using SynthSeg on T1‐weighted images, we obtained white matter parcellations and aggregated them to create time‐point‐specific BG and CSO ROI masks. To ensure consistency across time points, we limited the analysis to regions that were consistent across all time points. We then segmented PVS on T2‐weighted images using the RORPO filter followed by thresholding. All segmentations were visually assessed and manually corrected. We tested for differences using the Wilcoxon signed‐rank test.

**Result:**

PVS volumes measured at the three time points had high agreement with one another (Lin's concordance in BG ROI > 0.94 and in CSO ROI > 0.98).

Average BG‐PVS volumes at baseline were 133.38 mm^3^ [95%‐CI: 109.19,157.57]. Following acute exercise, these decreased to 123.10 mm^3^ [95%‐CI: 99.62,146.57], showing a significant reduction of 10.28 mm^3^ [95%‐CI: 3.24,17.33] (Figure 2; W=181, *p* = 0.003). After 24 hours, BG‐PVS volumes increased to 130.34 mm^3^ [95%‐CI: 107.96,152.72], similar to baseline levels (Figure 2; W=107, *p* = 0.644). CSO‐PVS volumes, on the other hand, showed no significant changes between baseline and after exercise or 24 hours later (Figure 2).

**Conclusion:**

Our work indicates that a single bout of physical exercise can exert subtle yet measurable volumetric changes on PVS in young participants. Whether this change reflects enhanced cerebrovascular or glymphatic function or not remains unclear, but will be explored in future research.